# Observations on the Nature and Cure of the Gout

**Published:** 1799-11

**Authors:** 


### *Obſervations on the Nature and Cure of the Gout.*

IN the fifth volume of the "*Medical Inquiries and Obſervations,*" by Dr. BENJAMIN RUSH, of Philadelphia, lately publiſhed, we meet with a number of ſtriking and original remarks on that Proteus-like diſorder. The conductors of the "Medical Repoſitory" having, in the third number of their ſecond volume, anticipated the account of this valuable work, we ſhall here avail ourſelves of their labours, and furniſh our readers with the following extracts on this ſubject.

So much has been offered to the public, concerning the Gout, by various authors, and at different times, that, if the actual developement of the diſeaſe had kept pace with the exertions made for this purpoſe, the ſubject might be now ſuppoſed to be nearly exhauſted; on the contrary, the unſucceſsful treatment of it has long been the reproach of medical imbecility; and whoever can throw any new light on this diſeaſe, will perform an acceptable ſervice to that large claſs of ſociety ſubjected to its tortures.

The author begins his obſervations on the gout, by delivering the following preliminary propoſitions: That it is a diſeaſe of the whole ſyſtem;—that it is a primary diſeaſe only of the ſolids;—that it affects fects most frequently persons of a sanguineous temperament; sometimes the nervous and phlegmatic, the idle and luxurious, rather than the temperate and laborious; and women as often as men, though commonly under feeble forms of morbid action, and not so often in their feet and limbs;—that its hereditary quality depends upon the propagation of a similar temperament from father to son;—and that it is always induced by general pre-disposing direct or indirect debility.

The remote causes of the gout, which induce this debility, are, indolence, great bodily labour, long protracted bodily exercise, intemperance in eating and in venery, acid aliments and drinks, strong tea and coffee, public and domestic vexation; the violent or long continued exercise of the understanding, imagination, and passions, in study, business, or pleasure; and lastly, the use of ardent and fermented liquors. The exciting causes of this disease are frequently a greater degree, or a sudden application of the remote and pre-disposing causes: To which may be added, sudden inanition from bleeding, purging, vomiting, and fasting; also cold, the debility left by the crisis of a fever; and all these aided by the additional direct debility induced upon the system by sleep; and the proximate cause is morbid excitement, accompanied with irregular action.

The gout affects most of the viscera. In the brain, it produces head-ach, vertigo, coma, apoplexy, and palsy. In the lungs, it produces pneumonia vera, notha, asthma, hæmoptysis, pulmonary consumption, and a short hecking cough. In the throat, it produces inflammatory angina. It affects the kidnies with inflammation, strangury, diabetes, and calculi. But of all the viscera, the liver suffers most from the gout; it produces in it inflammation, suppuration, melaina, scirrhus, gall-stones, jaundice, and an habitually increased secretion and excretion of bile.

The gout affects the alimentary canal from the stomach to the rectum. Flatulency, sickness, indigestion, pain, or vomitting, usually usher in a fit of the disease. Sick head-ach and dyspepsia are often the effects of this disease, concentrated in the stomach. The author asserts, he has seen a case in which the gout, by retreating to this viscus, produced the same burning sensation which is felt in the yellow fever. Cholic, dysentery, diarrhœa, piles, and a troublesome itching in the anus, are often produced by this disease.

This disease also affects the glands and lymphatics. It has been known to produce a ptyalism, and a bubo in the groin. The scrophula, and all the various forms of dropsy, have been observed as the effects of its disposition to attack the lymphatic system.

Cases of the gout affecting the skin have been often recorded: Erysipelas, gangrene, and petechiæ, are the acute; tetters and running sores the usual chronic forms in which it invades this part.

Even the bones are not exempted from the ravages of the gout. The bones of the hands and feet have been sometimes dislocated by it; and one instance occurred of its dislocating the thigh bone.

In the next place, the author proceeds to treat of the method of cure. The remedies for the gout naturally divide themselves into the following heads: 1. Such as are proper in its approaching or forming state. 2. Such as are proper in *violent* morbid actions in the blood-vessels and viscera. 3. Such as are proper in a *feeble* morbid action in the same parts of the body. 4. Such as are proper to relieve certain local symptoms not accompanied by general morbid action. And, 5. Such as are proper to prevent its recurrence, or to eradicate it from the system.

1. The symptoms of an approaching fit of the gout, are languor, dulness, doziness, giddiness, wakefulness, or sleep disturbed by vivid dreams; a dryness, and sometimes a coldness, numbness, and prickling in the feet and legs; occasional chills, acidity, and flatulency in the stomach; with an increased, a weak, or a defective appetite. During the existence of these symptoms, the disease may be easily prevented by the loss of a few ounces of blood, or by a gentle dose of physic; and afterwards by bathing the feet in warm water, by a few drops of the spirit of hartshorn, a draught of wine-whey, or an opiate. To these remedies, it is conceived, an emetic might be very properly added; as the affection of the stomach is so steadily observed at the approach of the disease, and as the analogy of fever would strictly warrant the practice.

2. Among the remedies proper in cases of great morbid action in the blood-vessels and viscera, our author chiefly relies upon blood-letting. He combats with decisive force the objections to this remedy, urged on the ground of the gout being a disease of debility, and that bleeding disposes to more frequent returns of it. He states the advantages of it to be the removal or lessening of pain;—the preventing of congestion and effusion in the more important viscera;—the preventing of the premature wearing out of the system, and the shortening of the duration of the fit. Among the remedies of this head, he also enumerates cathartics, emetics, nitre, cool or cold air, and sometimes cold water applied to to the inflamed part; the antiphlogistic regimen with respect to diet, blisters, and the operation of fear and terror. Cautions are suggested with respect to the use of sweating, opium, and several local applications. Blisters and caustics, as well as molasses, applied to the part affected, are much commended. The early exercise of the lower limbs, by walking, is also particularly enjoined.

3. The author next enumerates the remedies proper in that state of the gout in which a *feeble* morbid action takes place in the blood-vessels and viscera. They are opium, Madeira or sherry wine, porter, ardent spirits in the form of grog or toddy, æther, volatile alkali, aromatics, oil of amber, Peruvian bark, the warm bath, mercury given to the extent of ptyalism, and the local application of frictions with brandy and volatile liniment, blisters, and stimulating cataplasms.

4. The local symptoms of the gout are directed to be treated upon the common principles which are to be found in practical books.

5. The means of preventing the return of that state of the disease which is accompanied with violent action, are temperance, moderate labour and exercise, the avoiding of cold, moderation in the exercise of the understanding and passions, moderation in venereal indulgences, the avoiding of costiveness, and a stricter abstinence, as well as an attention to occasional evacuations by bleeding, &c. in the spring and autumn.

In the last place, the author mentions the remedies proper to obviate a return of that state of gout which is attended by a feeble morbid action in the blood-vessels and viscera. These are a gently stimulating diet; the use of chalybeate medicines; the volatile tincture of guajacum; the uniform application of warmth; the warm bath in winter, and the temperate or cold bath in summer; exercise; the avoiding of costiveness; the constant and agreeable employment of the understanding and passions, but without fatigue of body or mind; and, lastly, migration to a warmer climate.

We have thus delivered an outline of this valuable practical performance, in which the learned author has exploded much pernicious error, and introduced, illustrated, and forcibly enjoined, many important truths. The perusal, however, of the work itself, will afford a gratification which no reader can expect from this imperfect sketch.

